# Elevated Plasma Matrix Metalloproteinase 8 Associates With Sputum Culture Positivity in Pulmonary Tuberculosis^[Author-notes jiac160-FM2]^

**DOI:** 10.1093/infdis/jiac160

**Published:** 2022-05-01

**Authors:** N F Walker, F Karim, M Y S Moosa, S Moodley, M Mazibuko, K Khan, T R Sterling, Y F van der Heijden, A D Grant, P T Elkington, A Pym, A Leslie

**Affiliations:** Department of Clinical Sciences, Liverpool School of Tropical Medicine and Liverpool University Hospitals NHS Foundation Trust, Liverpool, United Kingdom; TB Centre and Department of Clinical Research, London School of Hygiene and Tropical Medicine, London, United Kingdom; Africa Health Research Institute, KwaZulu-Natal, South Africa; Department of Infectious Diseases, University of KwaZulu-Natal, Durban, South Africa; Africa Health Research Institute, KwaZulu-Natal, South Africa; Africa Health Research Institute, KwaZulu-Natal, South Africa; Africa Health Research Institute, KwaZulu-Natal, South Africa; Division of Infectious Diseases, Department of Medicine, Vanderbilt University Medical Center, Nashville, Tennessee, USA; Division of Infectious Diseases, Department of Medicine, Vanderbilt University Medical Center, Nashville, Tennessee, USA; The Aurum Institute, Johannesburg, South Africa; TB Centre and Department of Clinical Research, London School of Hygiene and Tropical Medicine, London, United Kingdom; Africa Health Research Institute, KwaZulu-Natal, South Africa; School of Laboratory Medicine and Medical Sciences, College of Health Sciences, University of KwaZulu-Natal, Durban, South Africa; NIHR Biomedical Research Centre, Clinical and Experimental Sciences, University of Southampton, Southampton, United Kingdom; Africa Health Research Institute, KwaZulu-Natal, South Africa; Africa Health Research Institute, KwaZulu-Natal, South Africa; School of Laboratory Medicine and Medical Sciences, College of Health Sciences, University of KwaZulu-Natal, Durban, South Africa; Division of Infection and Immunity, University College London, London, United Kingdom

**Keywords:** Tuberculosis, HIV, matrix metalloproteinase, procollagen III N-terminal propeptide, diagnosis, immunopathology

## Abstract

Current methods for tuberculosis treatment monitoring are suboptimal. We evaluated plasma matrix metalloproteinase (MMP) and procollagen III N-terminal propeptide concentrations before and during tuberculosis treatment as biomarkers. Plasma MMP-1, MMP-8, and MMP-10 concentrations significantly decreased during treatment. Plasma MMP-8 was increased in sputum *Mycobacterium tuberculosis* culture–positive relative to culture-negative participants, before (median, 4993 pg/mL [interquartile range, 2542–9188] vs 698 [218–4060] pg/mL, respectively; *P* = .004) and after (3650 [1214–3888] vs 720 [551–1321] pg/mL; *P* = .008) 6 months of tuberculosis treatment. Consequently, plasma MMP-8 is a potential biomarker to enhance tuberculosis treatment monitoring and screen for possible culture positivity.

## Teaser

In this cohort study, plasma matrix metalloproteinase 8 was increased in sputum culture–positive participants at tuberculosis diagnosis and after 6 months of tuberculosis treatment, demonstrating its potential as a biomarker of sputum culture positivity, to enhance tuberculosis treatment monitoring.

Tuberculosis is a major cause of disease and death worldwide, causing an estimated 9.9 million cases and approximately 1.5 million deaths in 2020, disproportionately affecting low-resource settings [1]. The treatment success rate for first-line tuberculosis treatment was only 86% in 2019. For people living with human immunodeficiency virus (HIV) and those with multidrug-resistant/rifampicin-resistant tuberculosis, treatment success rates are considerably lower (77% and 59%, respectively) [1].

Monitoring response to tuberculosis treatment is a challenge for national tuberculosis programs, especially in resource-limited settings. The World Health Organization recommends repeated sputum smear microscopy for acid-fast bacilli after 2 months of tuberculosis treatment for people with a new diagnosis of pulmonary tuberculosis receiving first-line treatment. Sputum culture for *Mycobacterium tuberculosis* drug susceptibility testing is reserved for cases in which sputum smear positivity persists or develops/recurs later in treatment [2]. Patients who are sputum smear-positive at diagnosis but smear-negative at month 2 are recommended to have repeated smears at the end of months 5 and 6. Both sputum smear and culture require appropriate laboratory facilities and trained personnel. Culture is limited by the considerable delay in availability of results. Poor specificity for viable organisms precludes use of molecular tests such as Xpert MTB/RIF for treatment monitoring [3]. Monitoring reliant on sputum production has diminished utility in sputum-nonproductive patients, including those too unwell, those with extrapulmonary tuberculosis, and those whose cough has resolved with treatment. The World Health Organization End TB strategy highlighted the need for new tools, including non–sputum-based diagnostics, to support effective patient-centered care [4].

Matrix metalloproteinases (MMPs) are host enzymes collectively capable of degrading the lung extracellular matrix at neutral pH. They are tightly regulated in vivo but have the potential to cause immunopathology [5]. MMP dysregulation is a keyfeature of tuberculosis immunopathology [6–10]. Sputum MMP-1 and MMP-8 are significantly elevated in patients with tuberculosis at diagnosis compared with controls, irrespective of HIV serostatus [7–9]. In plasma, MMP-1, MMP-8, and procollagen III N-terminal propeptide (PIIINP), a matrix degradation product released during collagen turnover, are elevated in patients with tuberculosis compared with healthy or respiratory symptomatic controls [8, 11]. In patients without HIV infection, elevated sputum MMP-1, MMP-2, MMP-3, and MMP-8 concentrations decrease after just 2 weeks of tuberculosis treatment [12]. In the current exploratory study, we evaluated plasma MMP and PIIINP concentrations and their association with sputum smear and culture status longitudinally in South African patients with tuberculosis, to determine the potential utility of MMPs and PIIINP as novel peripheral biomarkers of treatment response.

## METHODS

This was a retrospective analysis of the Collection of Sputum, Urine and Blood Samples for Research (CUBS) study (see also Supplementary Methods), which prospectively recruited adult participants in health facilities in eTheKwini municipality, KwaZulu-Natal, South Africa. Inclusion in this analysis required a diagnosis of tuberculosis (clinical and/or microbiological) leading to initiation of tuberculosis treatment. All CUBS study participants enrolled at the Prince Cyril Zulu Communicable Disease Centre (December 2013 to May 2014) were included. Plasma was collected at baseline (tuberculosis diagnosis) and visits at the end of months 2 (week 8) and 6 (week 24) of tuberculosis treatment. HIV testing was offered if the HIV status was unknown.

Sputum was collected for mycobacterial analysis, including smear, *M. tuberculosis* culture, and drug susceptibility testing at each visit. Culture was performed on solid (7H11) and liquid (MGIT) media. Plasma MMP-1, MMP-3, MMP-8, MMP-9, and MMP-10 were quantified by means of Luminex array (Bio-Rad Bio-Plex 200; assay from R&D Systems) and PIIINP by means of enzyme-linked immunosorbent assay (Cloud-Clone). The study was approved by University of KwaZulu-Natal and the London School of Hygiene & Tropical Medicine research ethics committees (references BE022/13 and 11710-1, respectively).

Analysis was performed using Prism 8 software (GraphPad). Comparisons between 2 groups were with the Mann-Whitney *U* test, and comparisons between multiple groups with the Kruskal-Wallis test and the Dunn multiple test comparison. Diagnostic accuracy was assessed with receiver operating characteristic (ROC) curve analysis, and associations between analytes with Spearman correlation.

## RESULTS

Participant (n = 85) characteristics at tuberculosis diagnosis are reported in Supplementary Tables 1 and 2. HIV serostatus was known to be positive in 43.5% (n = 37) and unknown in 11.8% (n = 10). The majority of participants were male (72.9% [n = 62]). Their median age was 35 years (IQR, 28.5–42.0 years; range, 18.0–60.0 years). Tuberculosis diagnosis was confirmed on sputum culture in 89.4% (n = 76). Baseline drug susceptibility testing results were available for 80% of participants (n = 68) and in 86.8% (n = 59) were fully sensitive.

### Plasma Collagenases and PIIINP Decrease During Tuberculosis Treatment

**Figure 1.**
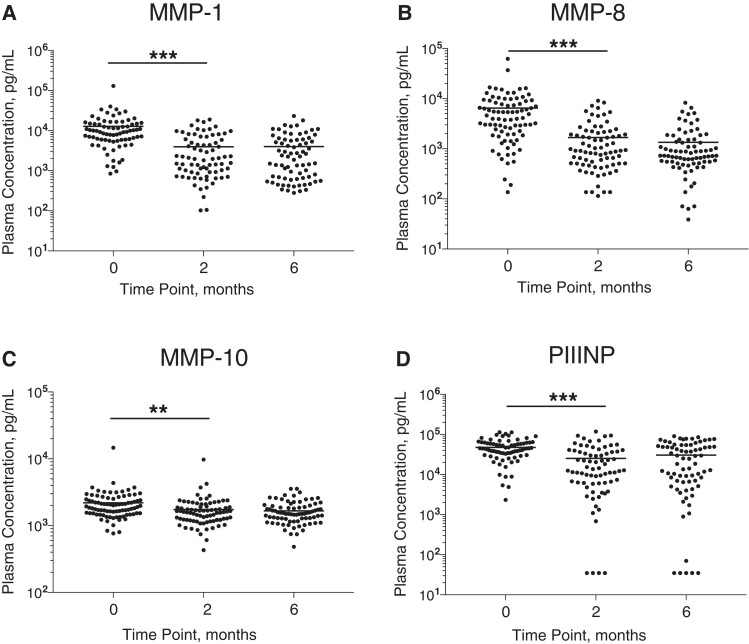
Plasma matrix metalloproteinase (MMP) 1, 8, and 10 and procollagen III N-terminal propeptide (PIIINP) concentrations during tuberculosis treatment. Plasma MMP-1 (*A*), MMP-8 (*B*), MMP-10 (*C*), and PIIINP (*D*) concentrations decreased between baseline (tuberculosis diagnosis) and the end of month 2 of tuberculosis treatment but did not decrease further between months 2 and 6. Analysis was by Kruskal-Wallis test with Dunn multiple test comparison. *P* values are summarised for the comparison between Time Point 0 and Time Point 2 months. **P < .01; ***P < .001.

The collagenases, MMP-1 (interstitial collagenase) and MMP-8 (neutrophil collagenase), decreased significantly between baseline and month 2, as did the stromelysin, MMP-10 (Figure 1*A*–1*C*). Concordantly, the matrix degradation product PIIINP, which is released during collagen turnover, decreased over the first 2 months of tuberculosis treatment (Figure 1*D*). No further significant reductions between month 2 and month 6 were observed. Conversely, plasma MMP-9 significantly increased between baseline and month 2, while plasma MMP-3 and MMP-7 did not significantly change (Supplementary Figure 1). Assessing correlations between analytes, including data from all time points, revealed a positive correlation between plasma PIIINP and the collagenases MMP-1 (*r* = 0.759; *P* < .001) and MMP-8 (*r* = 0.224; *P* < .001). MMP-1 and MMP-8 were also positively correlated (*r* = 0.377; *P* < .001). Full correlation results are reported in Supplementary Table 3.

### Elevated Plasma MMP-1 and PIIINP in Smear-Positive Disease

**Figure 2.**
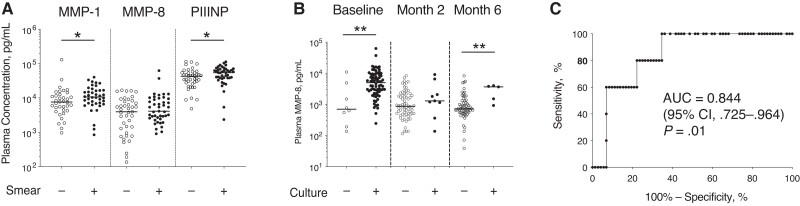
Plasma matrix metalloproteinase (MMP) 8 is increased in culture-positive tuberculosis at baseline and at month 6. *A,* Concentrations of plasma MMP-1 and procollagen III N-terminal propeptide (PIIINP), but not plasma MMP-8, were increased in participants who were smear-positive compared with those who were smear-negative at tuberculosis diagnosis. *B,* Plasma MMP-8 concentration was increased in *Mycobacterium tuberculosis* sputum culture–positive participants compared with culture-negative participants at tuberculosis diagnosis and at the end of month 6 of tuberculosis treatment. *C*, Receiver operating characteristic curve (ROC) analysis of plasma MMP-8 concentration 6 months after tuberculosis treatment initiation for identification of culture positivity at month 6. In *A* and *B,* analysis was by Mann-Whitney *U* test. *P < .05; **P < .01. (Where no *P* value is reported, *P* > .05.) Abbreviations: −, negative smear or culture; +, positive smear or culture; AUC, area under the ROC curve; CI, confidence interval.

Plasma MMPs and PIIINP were compared in sputum smear-positive and smear-negative participants at baseline (Figure 2*A*). MMP-1 and PIIINP were significantly increased in sputum smear-positive participants. MMP-3, MMP-8, MMP-9, and MMP-10 did not differ by smear status. A longitudinal analysis by smear status was not performed as only 1 participant had a subsequent smear-positive result during treatment.

### Plasma MMP-8 Associates With Sputum Culture Status at Tuberculosis Diagnosis and Month 6

At the end of month 6, five participants (5.8%) remained sputum culture-positive for *M. tuberculosis,* on liquid culture. Four of these 5 participants were HIV negative. *M. tuberculosis* culture confirmed that in 2 cases drug-sensitive isolates at diagnosis remained drug-sensitive at month 6, while in another 2 cases isolates that were isoniazid resistant at tuberculosis diagnosis were also rifampicin resistant at month 6, indicating the development of multidrug-resistant tuberculosis. In 1 case, drug susceptibility test results were available neither at diagnosis nor at later time points. Only 1 of these participants had a positive result on smear microscopy at month 6, and all participants were smear-negative at month 2, indicating that the majority of these cases would not have been identified by current microscopy-based methods of screening for treatment failure.

Plasma MMPs were compared in sputum culture–positive and culture-negative participants at each time point (Figure 2*B* and Supplementary Figure 2). Plasma MMP-8 was significantly increased in *M. tuberculosis* culture–positive compared with culture-negative participants at baseline (median [IQR], 4993 [2542–9188] vs 698 [281–4060] pg/mL, respectively; *P* = .004) and also at month 6 (3650 [1214–3888] vs 720 [551–1321] pg/mL; *P* = .008). However, no significant difference was found at month 2 (median [IQR], 1295 [754–4294] vs 870 [499–1986] pg/mL, respectively, for culture-positive vs culture-negative participants; *P* = .30). Analysis by HIV status was limited in power; however, a similar pattern of elevated MMP-8 associated with culture positivity at baseline and month 6 was seen in both HIV-negative and HIV-positive subgroups (Supplementary Table 4). There was a trend toward an association at month 2 in a subgroup analysis of male participants (Supplementary Figure 3). No other MMP concentration, nor PIIINP concentration, differed by sputum culture status at any time point. Plasma MMP-8 at month 6 predicted month 6 sputum culture status with an area under the curve of 0.844, corresponding to a sensitivity of 100% and a specificity of 65% at the optimal cutoff (>920 pg/mL) (Figure 2*C*).

## DISCUSSION

In this longitudinal analysis of patients with tuberculosis receiving treatment, we found that concentrations of plasma MMP-1, MMP-8, MMP-10, and PIIINP decreased with effective tuberculosis treatment over 2 months. While all but 1 participant in this study converted to smear-negative after 6 months of tuberculosis treatment, 5 participants were culture positive at 6 months. Elevated plasma MMP-8 at tuberculosis diagnosis and after 6 months of tuberculosis treatment was associated with sputum culture positivity, indicating that plasma MMP-8 is a candidate biomarker for monitoring treatment response.

Neutrophils are a potential source of MMP-8, which may be stored in granules before release. In vitro, neutrophils secrete MMP-8 directly in response to *M. tuberculosis* infection in a dose-dependent manner, and in response to cellular networks [13]. Our group has previously demonstrated that elevated plasma MMP-8 is associated with lipoarabinomannan positivity and neutrophil count in HIV-associated tuberculosis [8]. In patients starting tuberculosis treatment and then receiving antiretroviral therapy for HIV who go on to develop paradoxical tuberculosis–immune reconstitution inflammatory syndrome (IRIS), plasma MMP-8 is also increased at tuberculosis diagnosis and at tuberculosis-IRIS presentation [8]. Together, these findings suggest that plasma MMP-8 may be a surrogate plasma marker of mycobacterial load and neutrophil-driven immune responses in tuberculosis.

This study highlights the problem of identifying treatment failure in tuberculosis. Despite being started on tuberculosis treatment, 5 patients in the study remained culture positive for *M. tuberculosis* at 6 months. The majority of these would not have been identified by standard smear-based methods of assessing for treatment failure. A plasma biomarker, such as MMP-8, could provide a useful additional objective risk indicator to alert treating clinicians to the possibility of treatment failure, especially where resources are limited and in the case of sputum-nonproductive patients. If further developed for measurement using a low-cost point-of-care tool—for example, a lateral flow device—this could be implemented at the community level as a rule-out triage test, whereby a low reading supports treatment success and a high reading prompts repeated culture.

This study is not the first to identify an association of MMP-8 with culture positivity in patients receiving tuberculosis treatment. Sigal et al [14] reported an association of elevated ratios of serum MMP-8 at week 8 to baseline with culture positivity at weeks 8 and 12 but did not examine later time points. Lee et al [15] evaluated a number of potential biomarkers in plasma at baseline and at 2 months. At month 2, MMP-8 concentrations were increased in patients who were culture positive, compared with those who were culture negative, with an area under the ROC of 0.632 on ROC curve analysis. This is consistent with our findings, but at a different time point. Lee et al [15] included only participants with drug-sensitive tuberculosis, without HIV infection. Here, we report a cohort of patients of mixed HIV serostatus. The sample size limited our ability to perform subgroup analyses to explore the impact of HIV infection and antiretroviral therapy status on plasma MMP concentrations during tuberculosis treatment, and we did not evaluate the occurrence of tuberculosis-IRIS, but we hypothesize that these factors may influence plasma MMP concentrations, supported by findings in our previous study [8].

A strength of this cohort study was the detailed microbiological follow-up and inclusion of participants of mixed HIV serostatus, as well as drug-susceptible and drug-resistant tuberculosis cases. However, this was an exploratory study as opposed to a diagnostic accuracy assessment, and further evaluation is required to discern the clinical utility of these findings. The specificity of high plasma MMP-8 concentrations, especially in the context of other respiratory infections, requires further study. It is important to recognize that additional clinical factors, including symptoms and body mass index monitoring, may indicate patients in whom tuberculosis treatment is failing. We did not evaluate these indicators in this cohort. The sample size limited our ability to perform subgroup analyses, including in women and patients with drug-resistant tuberculosis, and we cannot exclude a role for unmeasured potential confounders (eg, smoking).

In conclusion, we describe an association of plasma MMP-8 with sputum *M. tuberculosis* culture positivity at the beginning and after 6 months of tuberculosis treatment, in a cohort of patients of mixed HIV serostatus. We advocate for the further evaluation of plasma MMP-8 as a biomarker of culture positivity to support tuberculosis treatment monitoring as a triage test, with the aim of early identification of treatment failure and appropriate allocation of diagnostic resources, to better support care of patients and improve tuberculosis treatment outcomes.

## Supplementary Material

jiac160_Supplementary_Data

## Supplementary Data

Supplementary materials are available at *The Journal of Infectious Diseases* online (http://jid.oxfordjournals.org/). Supplementary materials consist of data provided by the author that are published to benefit the reader. The posted materials are not copyedited. The contents of all supplementary data are the sole responsibility of the authors. Questions or messages regarding errors should be addressed to the author.

## Notes

***Acknowledgments.*** We thank Fernanda Maruri for assistance with study data collection.

***Financial support***. This work was supported by the British Infection Association; a National Institute for Health Research UK Starter Grant for Clinical Lecturers (Academy of Medical Sciences UK, Wellcome, Medical Research Council UK, British Heart Foundation, Arthritis Research UK, Royal College of Physicians, and Diabetes UK); the National Institutes of Health (grants U01 AI 069924 and K08 AI106420); the US Civilian Research and Development Foundation (grant OISE-16-62061-1); the South African Medical Research Council (grant RFA-CC: TB/HIV/AIDS-01-2014); Wellcome (210662/Z/18/Z to A. L.); and Medical Research Council UK (grant MR/P023754/1 to P. T. E.). Funding to pay the Open Access publication charges for this article was provided by Wellcome.
