# Supplementary material for: Elevated Plasma Matrix Metalloproteinase-8 associates with Sputum Culture Positivity in Pulmonary Tuberculosis
Source: J Infect Dis. Author manuscript; Available in PMC 2022 Sep 23. (PMC9470104; doi:10.1093/infdis/jiac160)
Supplement: Supplementary Material [file EMS146265-supplement-Supplementary_Material.docx]

# Supplement to:

# Elevated Plasma Matrix Metalloproteinase-8 associates with Sputum Culture Positivity in Pulmonary Tuberculosis

Walker N.F., Karim F., Moosa M.Y.S., Moodley S., Mazibuko M., Khan K., Sterling T.R., van der Heijden Y.F., Grant A.D., Elkington P.T., Pym A., Leslie A.

# Supplementary Methods

# The Collection of Sputum, Urine and Blood Samples (CUBS) for Research Study was a prospective cohort study which recruited participants in health facilities (hospitals, primary health care clinics, HIV counselling and testing sites, HIV treatment clinics, universities and other health care sites) in eTheKwini municipality, KwaZulu-Natal, South Africa. ETheKwini is one of 11 districts in the province and had a TB incidence of 685/100 000 (2015) and an antenatal clinic HIV prevalence of 41.1%. Eligibility for CUBS was determined by age 18 years or above and ability to give written informed consent.

# Sputum was spontaneous or induced with hypertonic saline, if required. Culture status was classified as “positive” if either solid or liquid culture was positive for *Mtb* complex, “negative” if both were negative or one was negative and the other not done or contaminated, and “indeterminate” if all sputum cultures were contaminated or no sputum was produced. Drug susceptibility testing (DST) was performed for isoniazid, rifampicin, ethambutol, streptomycin, ofloxacin and kanamycin. Plasma was collected in EDTA tubes at each timepoint. Participants were counselled on treatment adherence and attended the facilities regularly for adherence monitoring. No concerns about treatment adherence were recorded by the study team.

Plasma MMP-1, -3, -8, -9, and -10 were quantified by Luminex array (Bio-Rad Bio-Plex 200, assay from R&D Systems, UK) and PIIINP by ELISA (Cloud clone corp, China), according to manufacturer’s instructions.

# Supplementary Tables

## Supplementary Table S1 Participant demographic and clinical characteristics

Abbreviations: Interquartile range (IQR), Multidrug-resistant (MDR); *Mycobacterium tuberculosis (Mtb)*.

One patient with resistance to isoniazid and rifampicin also had resistance to aminoglycosides, ofloxacin and ethambutol.

## Supplementary Table S2 Clinical status of participants with HIV infection

| n | 37 |
| --- | --- |
| Most recent CD4, median cells/μL (IQR) | 198 (83.8-400) |
| On ART at enrolment, n (%) | 19 (51.4) |
| Undetectable HIV viral load result, if done (%) | 15 (40.5) |

Abbreviations: Antiretroviral therapy (ART), Interquartile range (IQR).

## Supplementary Table S3 Spearman r correlation between analytes

Abbreviations: Confidence interval (CI); Matrix metalloproteinase (MMP); Procollagen III N-terminal propeptide (PIIINP). Data were included from all timepoints.

To correct for multiple comparisons a p value <0.00238 was considered significant and these results are highlighted in bold.

## Supplementary Table S4 Median plasma MMP-8 concentrations by culture result and HIV serostatus

Values are median pg/ml and interquartile ranges (in brackets). P values are by Mann-Whitney U test. Not applicable (N/A) where frequency of one.

# Supplementary Figures

## Supplementary Figure S1 Plasma MMP-9, MMP-3 & MMP-7 concentrations during TB treatment

## Supplementary Figure S2 Plasma MMPs and PIIINP by sputum *Mycobacterium tuberculosis* culture status

## Supplementary Figure S3 Plasma MMP-8 by culture status in male participants

## Legend to Supplementary Figures

### Supplementary Figure S1 Plasma MMP-9, MMP-3 & MMP-7 concentrations during TB treatment

Plasma matrix metalloproteinase (MMP)-9 (A) increased between baseline (TB diagnosis) and month 2 following TB treatment initiation. Plasma MMP-3 (B) and MMP-7 (C) concentrations did not change over time with tuberculosis (TB) treatment. Analysis was by Kruskal-Wallis test with Dunn’s Multiple Test comparison. P values: ** p<0.001; where no p value is reported, p>0.05.

### Supplementary Figure S2 Plasma MMPs and PIIINP by sputum *Mycobacterium tuberculosis* culture status

Plasma MMP concentrations were compared by *Mycobacterium tuberculosis* culture status (positive + and negative -) at each timepoint. Analysis was by Mann-Whitney U test. P values are summarised: *p<0.05; ** p<0.001; where no p value is reported, p>0.05. Abbreviations: Matrix metalloproteinase (MMP); procollagen III N-terminal propeptide (PIIINP).

### Supplementary Figure S3 Plasma MMP-8 by culture status in male participants

Plasma MMP concentrations were compared by *Mycobacterium tuberculosis* culture status (positive + and negative -) at each timepoint in male participants only. Analysis was by Mann-Whitney U test. P values are summarised: *p<0.05; ** p<0.001 or stated. Abbreviations: Matrix metalloproteinase (MMP); procollagen III N-terminal propeptide (PIIINP).
